# Molecular Profile of Advanced Non-Small Cell Lung Cancers in Octogenarians: The Door to Precision Medicine in Elderly Patients

**DOI:** 10.3390/jcm8010112

**Published:** 2019-01-18

**Authors:** Caterina Fumagalli, Chiara Catania, Alberto Ranghiero, Carlo Bosi, Giuseppe Viale, Filippo de Marinis, Massimo Barberis, Elena Guerini-Rocco

**Affiliations:** 1 Unit of Histopathology and Molecular Diagnostics, Division of Pathology, IEO, European Institute of Oncology IRCCS, 20141 Milan, Italy; caterina.fumagalli@ieo.it (C.F.); alberto.ranghiero@ieo.it (A.R.); giuseppe.viale@ieo.it (G.V.); elena.GueriniRocco@ieo.it (E.G.-R.); 2 Division of Thoracic Oncology, IEO, European Institute of Oncology IRCCS, 20141 Milan, Italy; chiara.catania@ieo.it (C.C.); filippo.demarinis@ieo.it (F.d.M.); 3 Medical School, University of Milan, 20122 Milan, Italy; carlo.bosi@studenti.unimi.it; 4 Department of Oncology and Hemato-Oncology, University of Milan, 20122 Milan, Italy

**Keywords:** octogenarian, NSCLC, TKI therapy, NGS

## Abstract

Background: There is a pressing need to expand the evidence base in geriatric lung oncology. Most non-small cell lung cancers (NSCLCs) are diagnosed in the elderly, with approximately 15% of cases affecting octogenarians. Treatment-related decisions are challenging in this population, and the role of biologically driven therapies is still underrated. Methods: A single-institution cohort of 76 NSCLCs from octogenarian patients was submitted to molecular analysis using a next-generation sequencing (NGS) multigene panel, fluorescence in situ hybridization (FISH) analyses, and immunohistochemistry for PD-L1 assessment. Treatment and clinical outcome data were available for 33 patients. Results: Most cases (*n* = 66, 87%) harbored at least one genomic alteration. *EGFR* and *KRAS* mutations were detected in 18 (24%) and 20 (26%) patients, respectively. No *ALK* alterations were found, but in two patients *ROS1* translocation was identified. Of 22 cases tested, 17 were positive for PD-L1 staining. Octogenarian patients who received tyrosine kinase inhibitors (TKIs) based on molecular analysis showed clinical benefits, with long progression-free survival as expected in TKI-treated younger cohorts. Conclusions: This study highlights the utility of molecular profiling in all advanced-stage NSCLCs, regardless of the age at diagnosis, to drive personalized treatment. The prevalence of druggable alterations and the clinical benefits obtained by biologically-driven therapies in octogenarians were comparable to those of the younger NSCLC population.

## 1. Introduction

Lung cancer is the leading cause of cancer-specific mortality worldwide [1], and is typically a disease of the elderly. The median age at diagnosis is 70 years, and almost 10% of patients are older than 84 years [2]. Since the geriatric population has been increasing, the management of elderly patients affected by lung cancer is of growing concern [3]. Old patients with cancer usually have comorbidities and age-related physiological characteristics that make the choice of treatment challenging. Systemic cytotoxic chemotherapy, which has been the standard treatment of advanced lung cancer for decades, tends to be less-well-tolerated by elderly patients than by younger ones. The development of specific therapies targeting driver alterations (e.g., *EGFR* mutations or *ALK* and *ROS1* rearrangements) has changed the treatment paradigm and natural history of non-small cell lung cancer (NSCLC) harboring these aberrations [4,5]. To date, limited data are available regarding the safety and efficacy of these agents in the elderly population, and above all in octogenarian patients, since they are underrepresented in clinical trials [6,7]. Nevertheless, in clinical practice, the evaluation of tumor molecular features together with the clinical characteristics of octogenarian patients with NSCLC may broaden the treatment options and drive a tailored clinical management of these patients.

In the present study, we report the molecular characterization of advanced NSCLC from 76 consecutive octogenarian patients who were referred to our institution over 19 months for molecular diagnosis, following clinical requests. The molecular testing was performed using a next-generation sequencing (NGS) panel including *EGFR*, *KRAS*, and *BRAF* genes, in addition to fluorescence in situ hybridization (FISH) analyses for *ALK*, *ROS1*, *MET*, and immunohistochemical (IHC) evaluation of PD-L1 expression. We sought to (i) evaluate the biological characteristics of NSCLC occurring in octogenarian patients, which could lead to personalized treatments; and (ii) investigate the potential clinical benefit of molecularly driven therapies in a subgroup of patients treated with targeted treatment.

## 2. Materials and Methods

### 2.1. Patients

Of the 890 patients with advanced-stage (IIIb and IV) NSCLCs referred to the Molecular Diagnostics Unit of the European Institute of Oncology from August 2016 to February 2018, 76 patients over 80 years were included in this study. Molecular tests and IHC evaluation were required by thoracic oncologists, and included the analysis of *EGFR*, *KRAS*, *BRAF*, *ALK*, *ROS1*, *MET*, and PD-L1 according to the international guidelines for molecular testing in lung cancer [8,9]. Patients’ age, gender, smoking history, performance status (PS), and treatment regimens were collected from medical records and are summarized in Table 1. Based on their smoking status, patients were categorized as smokers, recent ex-smokers (more than 6 months but less than 15 years), long-term ex-smokers (more than 15 years), and never-smokers (Table 1). Thirty-three patients (43%) were inpatients and treated at the Division of Thoracic Oncology of the European Institute of Oncology. PS at diagnosis was evaluated according to the Eastern Cooperative Oncology Group (ECOG) criteria [10]. Patients’ treatments included chemotherapy (vinorelbine, pemetrexed, or gemcitabine), local radiotherapy, and anti-EGFR tyrosine kinase inhibitor (TKI) (erlotinib, gefitinib, or osimertinib) (Table 1). The best response to treatment was evaluated according to Response Evaluation Criteria in Solid Tumors (RECIST) [11].

All patients gave written informed consent regarding the storage of any biological specimens collected in the course of diagnosis and the use of these samples for research purposes.

### 2.2. Next-Generation Sequencing Analysis

Five-micrometer-thick sections from representative formalin-fixed paraffin-embedded (FFPE) tissue blocks (*n* = 63) and cytoblocks (*n* = 8) or smears (*n* = 5) were used for the analyses. The DNA was extracted automatically with the Promega Maxwell instrument (Promega, Madison, WI, USA) using the Promega Maxwell RSC DNA FFPE kit, and was quantified with the Quantus fluorometer (Promega, Madison, WI, USA). The NGS mutational analysis was performed with the CE-IVD (CE-marked, In-Vitro Diagnostics) Oncomine Solid Tumour DNA kit (ThermoFisher, Waltham, MA, USA). This panel allowed for the simultaneous evaluation of the mutational status (single-nucleotide variants, small insertions, and deletions) of 22 genes, namely *EGFR*, *ALK*, *ERBB2*, *ERBB4*, *FGFR1*, *FGFR2*, *FGFR3*, *MET*, *DDR2*, *KRAS*, *PIK3CA*, *BRAF*, *AKT1*, *PTEN*, *NRAS*, *MAP2K1*, *STK11*, *NOTCH1*, *CTNNB1*, *SMAD4*, *FBXW7*, and *TP53*. The targeted NGS analysis was performed following the manufacturer’s instructions. Briefly, 10 ng of genomic DNA was used for the library preparation. Sequencing was performed on a Personal Genome Machine (PGM) sequencer or Ion S5 System (ThermoFisher, Waltham, MA, USA), and data were analyzed using the Ion Reporter Analysis software v.5.2-5.10 (ThermoFisher, Waltham, MA, USA). Quality metric filters were used as previously described [12]. Only variants with an allele frequency ≥5% and those included in a public cancer gene mutation database (Cosmic: http://cancer.sanger.ac.uk/cosmic; ClinVar: https://www.ncbi.nlm.nih.gov/clinvar; TCGA Cancer Genome: http://cancergenome.nih.gov) were reported. Benign variants or polymorphisms were not reported. Mutations were classified as level I variants, level II variants, and level III variants according to their clinical relevance and/or availability of targeted therapy and the type of cancer under investigation [12,13,14,15].

### 2.3. Fluorescence in Situ Hybridization Analysis

*ALK* and *ROS1* gene rearrangements and *MET* amplification were evaluated by the standard FISH method. Briefly, unstained sections obtained from FFPE blocks or cytoblocks were incubated with an *ALK* and *ROS1* dual-color probe (IQFISH Break Apart Probe Agilent Technologies, Santa Clara, CA, USA). In each case, at least 100 tumor nuclei were evaluated. Cells were considered positive if a break-apart pattern of orange and green signals, at least one single orange signal, or a combination of both patterns were seen. Tumors with at least 15% of cells with *ALK* or *ROS1* rearrangements were defined as positive. In ambiguous or equivocal cases, ALK or ROS1 immunohistochemical stains (clone D5F3, Ventana, Tucson, AZ, USA and clone D4D6, Cell Signaling, Danvers, MA, USA, respectively) were performed. The presence of *MET* gene amplification was evaluated using the MET IQFISH Probe with CEP7 (Agilent Technologies, Santa Clara, CA, USA). Amplification was reported in cases with a MET Probe/CEP7 Ratio ≥2 and/or gene copy number ≥5.

### 2.4. PD-L1 Immunohistochemical Analysis

PD-L1 expression was evaluated on tumor cells using the immunohistochemistry assay CE-IVD PD-L1 IHC 22C3 pharmDx with the Agilent-Dako 22C3 clone developed on the Dako Autostainer Link 48 (Agilent Technologies, Santa Clara, CA, USA) as previously described [16]. The Tumor Proportion Score (TPS) was used to classify the cases as negative (IHC positive staining <1% neoplastic cells), 1%–49% positive cells, or >50% positive cells.

## 3. Results

### 3.1. Molecular Profile of Advanced Non-Small Cell Lung Cancer in Octogenarian Patients

**Figure 1:**
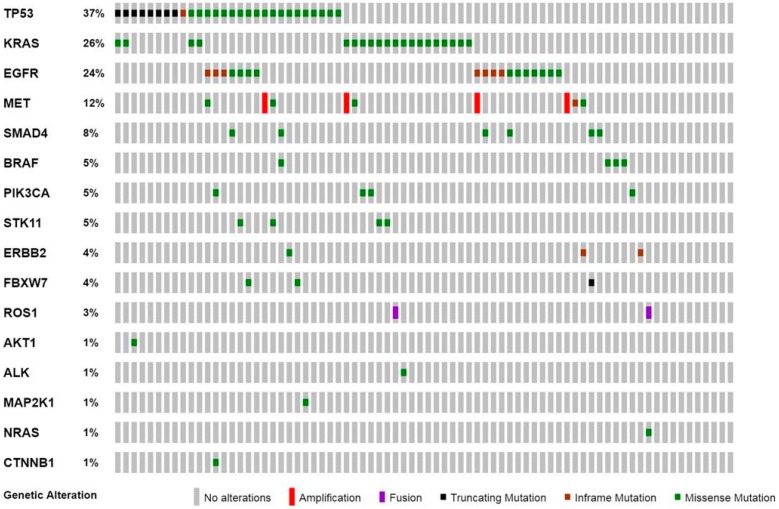
Oncoprint plot showing the distribution of genomic alterations identified in the study cohort. The co-occurrence of *EGFR* exon 19 deletion and T790M missense mutation was reported as a missense mutation. Genes without genomic alterations were not reported.

In our study population, 66 of 76 (87%) cases harbored at least one gene alteration, including clinically relevant and cancer-related variants. The most frequently mutated genes were *TP53* (37%), *KRAS* (26%), and *EGFR* (24%) (Figure 1 and Table 2).

Level I and level II alterations, defined by high clinical relevance for prognosis and/or target therapy available in a clinical setting (level I) or clinical trials (level II), accounted for 62/106 (58.5%) of genomic aberrations identified and involved *EGFR*, *ROS1*, *KRAS*, *BRAF* (1 case V600E), *MET*, *NRAS*, *ERBB2*, *PIK3CA*, and *AKT1* genes (Figure 1 and Table 2).

In detail, *KRAS* and *EGFR* mutations were reported in 20 and 18 NSCLC specimens, respectively. All *KRAS* mutations identified affected exon 2.

Mutations in *EGFR* included both TKI-sensitive mutations (*n* = 16), such as exon 19 deletions (*n* = 8) and exon 21 L858R point mutation (*n* = 8); and TKI-resistant mutations, such as exon 20 insertions (*n* = 2). In four cases, a secondary T790M resistant mutation was detected during TKI treatment. Concurrent *EGFR*, T790M, and L858R mutations were present at diagnosis in one patient with no history of TKI therapy.

No *ALK* fusions were detected in our study cohort. *ROS1* gene rearrangements were identified in two cases, with a concurrent *NRAS* mutation or *KRAS* mutation.

Moreover, *MET* alterations were found in 12% of cases and included missense variants (*n* = 4), exon 14 skipping mutations (*n* = 1), and gene amplifications (*n* = 4).

Overall concurrent genetic aberrations were found in 33 cases (Figure 1), including *TP53* mutations with either *EGFR* (*n* = 7) or *KRAS* (*n* = 4) mutations. Moreover, *EGFR* mutations were found in association with alterations of *MET* (*n* = 2) and *STK11* (*n* = 1) genes. Concurrent *KRAS* and *STK11* mutations were identified in two cases.

PD-L1 evaluation was requested for a subgroup of octogenarian patients (*n* = 22, 29%), including five negative cases (25%), seven cases (25%) with intermediate TPS between 1% and 49%, and ten cases (50%) with diffuse PD-L1 expression (TPS > 50%) (Table 2). Among the 17 positive cases, five had concurrent *EGFR* mutations, five had concurrent *KRAS* mutations, and two had concurrent *BRAF* mutations.

### 3.2. Treatment Regimens and Clinical Outcome of Octogenarian Patients with Advanced Non-Small Cell Lung Cancers

Treatment and follow-up data were available for thirty-three inpatients. Twenty-three of these patients received an active treatment (Table 2). In ten cases, only radiotherapy or best supportive care was given because of the presence of comorbidities or poor performance status, including four patients with a PS of two, five patients with a PS of three, and one patient with a PS of one who had previously undergone right nephrectomy for renal cell carcinoma. Data about targeted treatments were available only for patients with *EGFR* mutations. The other actionable alterations (in *ROS1*, *ERBB2*, *MET*, and *BRAF*) were found in NSCLC outpatients or inpatients with a poor PS, not eligible for enrollment in a clinical trial or active treatment.

**Figure 2:**
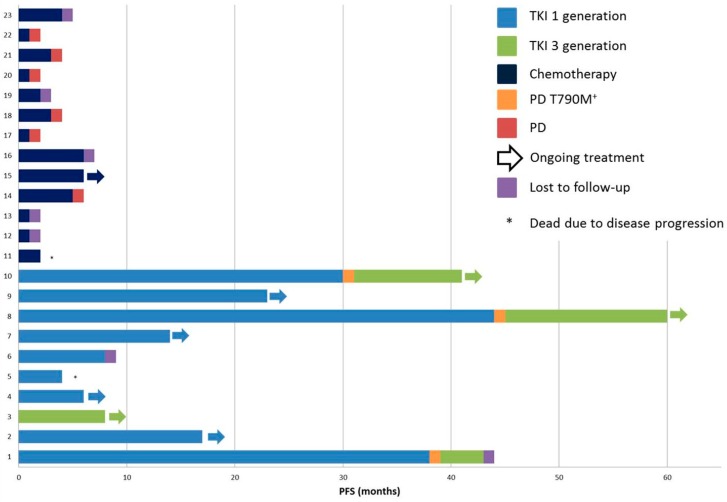
Progression-free survival (PFS) of patients actively treated with chemotherapy and tyrosine kinase inhibitors (TKIs).

Among the patients with *EGFR* mutations, the majority were never-smokers or long-term ex-smokers (*n* = 13/18 cases, 72.2%). Smoking habits were not assessed in the remaining cases. A different distribution between males and females was not seen (8 males and 12 females). Treatment and follow-up data were available for ten patients who received tyrosine kinase inhibitors (TKIs), including erlotinib, gefitinib, or osimertinib, when a concurrent *EGFR* T790M mutation was detected. One patient was first-line treated with osimertinib because of the co-occurrence of L858R and T790M mutations at diagnosis (patient #3, Figure 2 and Table 3). The median progression-free survival (PFS) for patients receiving TKI as first-line therapy was 17 months, including five patients in whom treatment was ongoing (Figure 2). One patient died of progressive disease after 4 months of treatment. During first-line TKI therapy, two patients developed the *EGFR* T790M resistance mutation and received osimertinib as second-line treatment. The best responses observed were complete responses in two patients (#7 and #8, Figure 2), reached after four months of gefitinib and 5 months of osimertinib, respectively. Both patients were still receiving treatment after 14 months of gefitinib and 15 months of osimertinib, respectively.

Thirteen patients received chemotherapy-based regimens, including metronomic vinorelbine (*n* = 10) or platinum-based therapy (*n* = 3). The median PFS of these patients was 2 months (Figure 2).

## 4. Discussion

Treatment-related decisions in octogenarians affected by NSCLC are challenging: the elderly could have reduced tolerance of treatments because of limited physiological reserve or multiple comorbid conditions, but they could also be under-treated based solely on biological age [6]. Case-by-case clinical assessment in addition to tumor molecular analyses could lay the basis for tailored treatment choices. However, few data are available regarding the real-life management of octogenarian patients with NSCLC.

In the present study, we focused on a single-institution cohort of 76 consecutive octogenarian patients affected by advanced NSCLC. All tumors were analyzed using an NGS multi-gene panel that expands the tumor genetic portrait, including genes such as *BRAF*, *ERBB2*, *KRAS*, and *MET* in addition to *EGFR* and *ALK*/*ROS1*, in accordance with recently updated recommendations [8,9]. Most cases (86.8%) harbored at least one genetic alteration, and in only ten cases no genomic aberration was detected in the genes tested. These data were consistent with our recent findings obtained in a larger cohort of NSCLC patients, not selected by age at diagnosis [12]. Indeed, among 535 NSCLC patients, we found that 82.4% harbored at least one NGS-detected mutation, compared to 85.5% within this study cohort. In the present study, we reported level I and II variants in 65.8% of the cases, including *EGFR* (24%) and *KRAS* (26%) mutations. In our previous series, level I and II variants were detected in 63.6% of cases, with *KRAS* and *EGFR* mutations present in 31% and 22% of the cases, respectively [12]. The differences in mutation distribution between the two series were not statistically significant (^2^ test *p* > 0.05) and showed that the presence of specific tumor genetic aberrations was not related to the age of the patients. Conversely, *ALK* fusions were not detected in our cohort, consistent with the reported association between *ALK* translocation and the patients’ younger age [17,18]. Surprisingly, we found *ROS1* translocations in two patients. This rarest druggable alteration has been reported in 1%–2% of NSCLCs [19,20], usually affecting never-smoker young female patients [19]. In our cohort, two octogenarian males harbored a *ROS1* translocation, and both had concurrent clinically relevant mutations (*ROS1*^+^/*KRAS*^+^ and *ROS1*^+^/*NRAS*^+^). Similar anecdotal cases of concurrent *ROS1* rearrangements and *KRAS* mutations were recently reported [21,22], whereas no cases of *ROS1* translocations and *NRAS* variants have been described so far. *ROS1* gene rearrangements could be targeted by TKIs, but *KRAS*/*NRAS* gene point mutations could lead to ineffective inhibition through inactivation of the RAF/MEK/ERK signaling pathway. Given that these patients were both outpatients, no data about therapeutic regimens or clinical outcomes were available.

In a subgroup of octogenarian patients, we also identified alterations in *MET*, *BRAF*, and *ERBB2* genes, which may be targeted by specific therapies in a clinical setting or clinical trials [23,24]. Moreover, we reported level III mutations that could be clinically significant. *TP53* mutations have recently been associated with prognosis or therapy response in patients with concomitant *EGFR* or *KRAS* mutations, or *ALK* rearrangement [25]. Patients whose tumors harbored concurrent *EGFR* and *TP53* mutations showed a worse response rate to TKIs and worse progression-free survival [26]. *KRAS^+^-TP53^+^* tumors displayed a higher level of inflammatory markers and improved relapse-free survival [27].

In the octogenarians included in this study, *EGFR* mutated status was associated with non-smoking, as in the general population of NSCLC patients. A TKI treatment was administered to ten *EGFR* mutation-positive patients, who showed a PFS consistent with data from larger series of NSCLC patients [28,29,30,31,32,33,34,35]. Although these series included patients ranging from 24 to 88 years old, the elderly were underrepresented and specific data regarding octogenarians are limited. Previous studies have reported on the safety and efficacy of TKIs in elderly patients, as shown by osimertinib treatment for T790M-positive NSCLC [36] or erlotinib treatment for NSCLC after one or more platinum-based chemotherapy regimens [37]. However, the definition of “elderly” is highly heterogeneous among these studies, including patients ranging from 65 [37] to 75 years old [36]. Recently, a multicenter real-world study (OCTOMUT) evaluated the tolerability and efficacy of TKIs in Caucasian octogenarian patients with *EGFR*-mutated NSCLC [38]. The authors reported clinical outcomes and toxicity profiles comparable to those in younger patients. The median PFS observed in that retrospective study was 11.9 months for patients treated with TKIs. Although the number of patients with follow-up data was limited in our cohort, we observed a prolonged response in six of ten patients treated with TKI therapy. Moreover, seven patients are receiving ongoing treatment.

During the past few years, the spectrum of therapeutic options for NSCLC patients with no identified genomic aberrations has been enlarged. Besides standard chemotherapy regimens, treatments with immune checkpoint inhibitors alone or in combination with chemotherapy have been shown to be effective in selected patients with advanced NSCLC enrolled in clinical trials. However, in the largest series, elderly patients were underrepresented, especially octogenarians [39]. The ELDERS Study is a prospective pilot study evaluating the role of checkpoint inhibitors in patients ≥70 years old [40]. Although there are some promising results, the real benefit of immunotherapy in this specific population is still under debate. In this study population, only a few tumors were tested for tumor PD-L1 expression (the only approved biomarker to select patients for immunotherapy in our country) and were found to have the whole range of TPS scores. Retrospective collections of clinical data from a large series of elderly individuals who received immunotherapies and, above all, prospective studies focused on NSCLC patients ≥80 years old are warranted in order to clarify the role of immunotherapy in the octogenarian population.

A major limitation of the present study is the relatively small cohort of patients analyzed. However, we collected consecutive octogenarian patients affected by NSCLC who underwent tumor molecular analyses for diagnostic purposes in a single institution. Another limitation is that treatment and follow-up data were not available for all patients and were collected retrospectively from patients’ medical records. We reported data for thirty-three inpatients of the Division of Thoracic Oncology of the European Institute of Oncology, including twenty-three patients who received molecularly driven treatments. Although the small cohort prevented identification of any clinical or statistical correlations and the retrospective analysis was not selected for the probability of survival, we reported interesting insights into the clinical course of octogenarian patients actively treated based on tumor molecular characteristics.

## 5. Conclusions

This study highlights the utility of molecular analyses for all patients with advanced-stage NSCLC, regardless of age at diagnosis, to guide clinical decisions. Except for ALK translocation, the distribution of molecular alterations in the octogenarian population under investigation was very similar to that in a younger population. Molecular analyses should be performed with multi-gene panels, following the recent recommendations for molecular testing in NSCLC [8,9], to widen the number of predictive markers that may be detected. Indeed, most of the NSCLCs analyzed in this study harbored at least one actionable or clinically-relevant gene alteration. This information may guide clinical decisions about active treatments for these patients with clinical benefits. In the era of precision medicine, age itself may not represent a contraindication to evaluate the molecular characteristics of a tumor. Rather, a comprehensive assessment of clinical, pathological, and molecular features of octogenarian patients with NSCLC may improve the selection of tailored and effective treatments for this population.

## Figures and Tables

**Table 1:** Clinicopathological characteristics of patients included in the study.

Age	Median (Range)
≥80 years old	82 (80–92)
**Gender**	***n* (%)**
Male	58 (76.3%)
Female	18 (23.7%)
**Histology**	***n* (%)**
Adenocarcinoma	66 (86.8%)
Adenosquamous carcinoma	2 (2.6%)
Non-squamous NSCLC	8 (10.6%)
**Smoking Habit**	***n* (%)**
Smokers	6 (7.9%)
Recent ex-smokers	14 (18.4%)
Long-term ex-smokers	16 (21.1%)
Never-smokers	18 (23.7%)
NA	22 (28.9%)
**Performance Status (PS)**	***n* (%)**
PS = 0	1 (1.3%)
PS = 1	20 (26.3%)
PS = 2	11 (14.5%)
PS > 2	5 (6.6%)
NA	39 (51.3)
**Treatment Regimens**	***n* (%)**
Chemotherapy	13 (17%)
Tyrosine kinase inhibitor	10 (13.2%)
Radiotherapy and/or best supportive care	10 (13.2%)
NA	43 (56.6%)

NA: data not available.

**Table 2:** Frequencies of genomic alterations and PD-L1 expression detected in NSCLCs from the octogenarian patients of the study.

**Gene (No. of cases tested = 76)**	***n* (No. of cases)**
*EGFR* mutation	18
*ALK* rearrangement	0
*ROS1* rearrangement	2
*KRAS* mutation	20
*MET* mutation/amplification	9
*BRAF* mutation	4
*ERBB2* mutation	3
Alterations in other genes	22
All genes wild-type	10
**PD-L1 IHC (N. of cases tested = 22)**	***n***
TPS < 1%	5
TPS = 1%–49%	7
TPS > 50%	10

IHC: immunohistochemistry; TPS: Tumor Proportion Score.

**Table 3:** Clinical and molecular characteristics of the 23 inpatients who received active treatment.

Patient #	Mutational Status	PD-L1 TPS	Performance Status	Treatment
1	EGFR p.Glu746_Ala750del EGFR p.Thr790Met	>50%	PS 0	TKIs—Gefitinib and Osimertinib
2	EGFR p.Glu746_Ala750del MET amplified	>50%	PS 1	TKI—Gefitinib
3	EGFR p.Leu858Arg EGFR p.Thr790Met TP53 p.Asn131Tyr	1%–49%	PS 1	TKI—Osimertinib
4	EGFR p.Leu858Arg TP53 p.Pro80Ser	NA	PS 1	TKI—Gefitinib
5	EGFR p.Leu858Arg	NA	PS 2	TKI—Gefitinib
6	EGFR p.Glu746_Ala750del	1%–49%	PS 1	TKI—Gefitinib
7	EGFR p.Leu858Arg	NA	PS 1	TKI—Gefitinib
8	EGFR p.Glu746_Thr752delinsAla EGFR p.Thr790Met TP53 p.Cys176Tyr SMAD4 p.Phe354Leu	NA	PS 1	TKIs—Erlotinib and Osimertinib
9	EGFR p.Leu858Arg TP53 p.Val157Phe STK11 p.Phe354Leu	NA	PS 1	TKI—Gefitinib
10	EGFR p.Glu746_Ala750del EGFR p.Thr790Met	NA	PS 1	TKIs—Erlotinib and Osimertinib
11	WT	NA	PS 1	Chemotherapy—Vinorelbine
12	WT	<1%	PS 2	Chemotherapy—Combination regimens *
13	WT	<1%	PS 1	Chemotherapy—Vinorelbine
14	KRAS p.Gly12Val	<1%	PS 2	Chemotherapy—Vinorelbine
15	TP53 p.Asn131Tyr	NA	PS 1	Chemotherapy—Vinorelbine
16	SMAD4 p.Gln256Leu	NA	PS 1	Chemotherapy—Combination regimens *
17	PIK3CA p.Glu545Lys	NA	PS 1	Chemotherapy—Vinorelbine
18	KRAS p.Gly12Cys PIK3CA p.Glu545Lys	NA	PS 2	Chemotherapy—Vinorelbine
19	KRAS p.Gly12Cys TP53 Glu349Ter	<1%	PS 2	Chemotherapy—Vinorelbine
20	KRAS p.Gly12Asp TP53 p.Val216Met	1%–49%	PS 1	Chemotherapy—Vinorelbine
21	KRAS p.Gly12Val STK11 p.Asp194Tyr	NA	PS 2	Chemotherapy—Vinorelbine
22	KRAS p.Gly13Cys STK11 p.Arg331Trp	>50%	PS 2	Chemotherapy—Vinorelbine
23	TP53 p.Gys275Phe ERBB2 p.Ile767Met	NA	PS 1	Chemotherapy—Combination regimens *

#: Patient’s ID; TPS: Tumor Proportion Score; PS: performance status; TKI: tyrosine kinase inhibitor; WT: wild-type; NA: data not available; *: combination regimens including gemcitabine-pemetrexed-carboplatin.

## References

[1] Siegel R., Ma J., Zou Z., Jemal A. (2014). Cancer statistics, 2014. Cancer J. Clin..

[2] Bethesda SEER Cancer Stat Facts: Lung and Bronchus Cancer. National Cancer Institute. https://seer.cancer.gov/statfacts/html/lungb.html.

[3] Gonzalez-Aragoneses F., Moreno-Mata N., Simon-Adiego C., Penalver-Pascual R., Gonzalez-Casaurran G., Perea L.A. (2009). Lung cancer surgery in the elderly. Crit. Rev. Oncol. Hematol..

[4] Vestergaard H.H., Christensen M.R., Lassen U.N. (2018). A systematic review of targeted agents for non-small cell lung cancer. Acta Oncol..

[5] Ryser C.O., Diebold J., Gautschi O. (2019). Treatment of anaplastic lymphoma kinase-positive non-small cell lung cancer: Update and perspectives. Curr. Opin. Oncol..

[6] Wang S., Wong M.L., Hamilton N., Davoren J.B., Jahan T.M., Walter L.C. (2012). Impact of age and comorbidity on non-small-cell lung cancer treatment in older veterans. J. Clin. Oncol..

[7] Schulkes K.J., Nguyen C., van den Bos F., van Elden L.J., Hamaker M.E. (2016). Selection of Patients in Ongoing Clinical Trials on Lung Cancer. Lung.

[8] Kalemkerian G.P., Narula N., Kennedy E.B. (2018). Molecular Testing Guideline for the Selection of Lung Cancer Patients for Treatment with Targeted Tyrosine Kinase Inhibitors: American Society of Clinical Oncology Endorsement Summary of the College of American Pathologists/International Association for the Study of Lung Cancer/Association for Molecular Pathology Clinical Practice Guideline Update. J. Oncol. Pract..

[9] Lindeman N.I., Cagle P.T., Aisner D.L., Arcila M.E., Beasley M.B., Bernicker E.H., Colasacco C., Dacic S., Hirsch F.R., Kerr K. (2018). Updated Molecular Testing Guideline for the Selection of Lung Cancer Patients for Treatment with Targeted Tyrosine Kinase Inhibitors: Guideline From the College of American Pathologists, the International Association for the Study of Lung Cancer, and the Association for Molecular Pathology. J. Thorac. Oncol..

[10] Oken M.M., Creech R.H., Tormey D.C., Horton J., Davis T.E., McFadden E.T., Carbone P.P. (1982). Toxicity and response criteria of the Eastern Cooperative Oncology Group. Am. J. Clin. Oncol..

[11] Eisenhauer E.A., Therasse P., Bogaerts J., Schwartz L.H., Sargent D., Ford R., Dancey J., Arbuck S., Gwyther S., Mooney M. (2009). New response evaluation criteria in solid tumours: Revised RECIST guideline (version 1.1). Eur. J. Cancer.

[12] Fumagalli C., Vacirca D., Rappa A., Passaro A., Guarize J., Rafaniello Raviele P., de Marinis F., Spaggiari L., Casadio C., Viale G. (2018). The long tail of molecular alterations in non-small cell lung cancer: A single-institution experience of next-generation sequencing in clinical molecular diagnostics. J. Clin. Pathol..

[13] Richards S., Aziz N., Bale S., Bick D., Das S., Gastier-Foster J., Grody W.W., Hegde M., Lyon E., Spector E. (2015). Standards and guidelines for the interpretation of sequence variants: A joint consensus recommendation of the American College of Medical Genetics and Genomics and the Association for Molecular Pathology. Genet. Med..

[14] Li M.M., Datto M., Duncavage E.J., Kulkarni S., Lindeman N.I., Roy S., Tsimberidou A.M., Vnencak-Jones C.L., Wolff D.J., Younes A. (2017). Standards and Guidelines for the Interpretation and Reporting of Sequence Variants in Cancer: A Joint Consensus Recommendation of the Association for Molecular Pathology, American Society of Clinical Oncology, and College of American Pathologists. J. Mol. Diagn..

[15] Sukhai M.A., Craddock K.J., Thomas M., Hansen A.R., Zhang T., Siu L., Bedard P., Stockley T.L., Kamel-Reid S. (2016). A classification system for clinical relevance of somatic variants identified in molecular profiling of cancer. Genet. Med..

[16] Marchetti A., Barberis M., Franco R., De Luca G., Pace M.V., Staibano S., Volante M., Buttitta F., Guerini-Rocco E., Righi L. (2017). Multicenter Comparison of 22C3 PharmDx (Agilent) and SP263 (Ventana) Assays to Test PD-L1 Expression for NSCLC Patients to Be Treated with Immune Checkpoint Inhibitors. J. Thorac. Oncol..

[17] Kwak E.L., Bang Y.J., Camidge D.R., Shaw A.T., Solomon B., Maki R.G., Ou S.H., Dezube B.J., Janne P.A., Costa D.B. (2010). Anaplastic lymphoma kinase inhibition in non-small-cell lung cancer. N. Engl. J. Med..

[18] Inamura K., Takeuchi K., Togashi Y., Hatano S., Ninomiya H., Motoi N., Mun M.Y., Sakao Y., Okumura S., Nakagawa K. (2009). EML4-ALK lung cancers are characterized by rare other mutations, a TTF-1 cell lineage, an acinar histology, and young onset. Mod. Pathol..

[19] Bergethon K., Shaw A.T., Ou S.H., Katayama R., Lovly C.M., McDonald N.T., Massion P.P., Siwak-Tapp C., Gonzalez A., Fang R. (2012). ROS1 rearrangements define a unique molecular class of lung cancers. J. Clin. Oncol..

[20] Davies K.D., Le A.T., Theodoro M.F., Skokan M.C., Aisner D.L., Berge E.M., Terracciano L.M., Cappuzzo F., Incarbone M., Roncalli M. (2012). Identifying and targeting ROS1 gene fusions in non-small cell lung cancer. Clin. Cancer Res..

[21] Lin J.J., Ritterhouse L.L., Ali S.M., Bailey M., Schrock A.B., Gainor J.F., Ferris L.A., Mino-Kenudson M., Miller V.A., Iafrate A.J. (2017). ROS1 Fusions Rarely Overlap with Other Oncogenic Drivers in Non-Small Cell Lung Cancer. J. Thorac. Oncol..

[22] Zhu Y.C., Lin X.P., Li X.F., Wu L.X., Chen H.F., Wang W.X., Xu C.W., Shen J.F., Wei J.G., Du K.Q. (2018). Concurrent ROS1 gene rearrangement and KRAS mutation in lung adenocarcinoma: A case report and literature review. Thorac. Cancer.

[23] Li B.T., Shen R., Buonocore D., Olah Z.T., Ni A., Ginsberg M.S., Ulaner G.A., Offin M., Feldman D., Hembrough T. (2018). Ado-Trastuzumab Emtansine for Patients With HER2-Mutant Lung Cancers: Results From a Phase II Basket Trial. J. Clin. Oncol..

[24] Baik C.S., Myall N.J., Wakelee H.A. (2017). Targeting BRAF-Mutant Non-Small Cell Lung Cancer: From Molecular Profiling to Rationally Designed Therapy. Oncologist.

[25] Costa D.B. (2018). TP53 mutations are predictive and prognostic when co-occurring with ALK rearrangements in lung cancer. Ann. Oncol..

[26] VanderLaan P.A., Rangachari D., Mockus S.M., Spotlow V., Reddi H.V., Malcolm J., Huberman M.S., Joseph L.J., Kobayashi S.S., Costa D.B. (2017). Mutations in TP53, PIK3CA, PTEN and other genes in EGFR mutated lung cancers: Correlation with clinical outcomes. Lung Cancer.

[27] Skoulidis F., Byers L.A., Diao L., Papadimitrakopoulou V.A., Tong P., Izzo J., Behrens C., Kadara H., Parra E.R., Canales J.R. (2015). Co-occurring genomic alterations define major subsets of KRAS-mutant lung adenocarcinoma with distinct biology, immune profiles, and therapeutic vulnerabilities. Cancer Discov..

[28] Haspinger E.R., Agustoni F., Torri V., Gelsomino F., Platania M., Zilembo N., Gallucci R., Garassino M.C., Cinquini M. (2015). Is there evidence for different effects among EGFR-TKIs? Systematic review and meta-analysis of EGFR tyrosine kinase inhibitors (TKIs) versus chemotherapy as first-line treatment for patients harboring EGFR mutations. Crit. Rev. Oncol. Hematol..

[29] Rosell R., Carcereny E., Gervais R., Vergnenegre A., Massuti B., Felip E., Palmero R., Garcia-Gomez R., Pallares C., Sanchez J.M. (2012). Erlotinib versus standard chemotherapy as first-line treatment for European patients with advanced EGFR mutation-positive non-small-cell lung cancer (EURTAC): A multicentre, open-label, randomised phase 3 trial. Lancet Oncol..

[30] Mok T.S., Wu Y.L., Thongprasert S., Yang C.H., Chu D.T., Saijo N., Sunpaweravong P., Han B., Margono B., Ichinose Y. (2009). Gefitinib or carboplatin-paclitaxel in pulmonary adenocarcinoma. N. Engl. J. Med..

[31] Sequist L.V., Yang J.C., Yamamoto N., O’Byrne K., Hirsh V., Mok T., Geater S.L., Orlov S., Tsai C.M., Boyer M. (2013). Phase III study of afatinib or cisplatin plus pemetrexed in patients with metastatic lung adenocarcinoma with EGFR mutations. J. Clin. Oncol..

[32] Soria J.C., Ohe Y., Vansteenkiste J., Reungwetwattana T., Chewaskulyong B., Lee K.H., Dechaphunkul A., Imamura F., Nogami N., Kurata T. (2018). Osimertinib in Untreated EGFR-Mutated Advanced Non-Small-Cell Lung Cancer. N. Engl. J. Med..

[33] Janne P.A., Yang J.C., Kim D.W., Planchard D., Ohe Y., Ramalingam S.S., Ahn M.J., Kim S.W., Su W.C., Horn L. (2015). AZD9291 in EGFR inhibitor-resistant non-small-cell lung cancer. N. Engl. J. Med..

[34] Mok T.S., Wu Y.-L., Ahn M.-J., Garassino M.C., Kim H.R., Ramalingam S.S., Shepherd F.A., He Y., Akamatsu H., Theelen W.S. (2017). Osimertinib or Platinum-Pemetrexed in EGFR T790M-Positive Lung Cancer. N. Engl. J. Med..

[35] Hutchinson L. (2017). Lung cancer: AURA3 magic reveals new standard. Nat. Rev. Clin. Oncol..

[36] Furuta H., Uemura T., Yoshida T., Kobara M., Yamaguchi T., Watanabe N., Shimizu J., Horio Y., Kuroda H., Sakao Y. (2018). Efficacy and Safety Data of Osimertinib in Elderly Patients with NSCLC Who Harbor the EGFR T790M Mutation After Failure of Initial EGFR-TKI. Treat. Anticancer Res..

[37] Brueckl W.M., Achenbach H.J., Ficker J.H., Schuette W. (2018). Erlotinib treatment after platinum-based therapy in elderly patients with non-small-cell lung cancer in routine clinical practice—Results from the ElderTac study. BMC Cancer.

[38] Corre R., Gervais R., Guisier F., Tassy L., Vinas F., Lamy R., Fraboulet G., Greillier L., Doubre H., Descourt R. (2018). Octogenarians with EGFR-mutated non-small cell lung cancer treated by tyrosine-kinase inhibitor: A multicentric real-world study assessing tolerance and efficacy (OCTOMUT study). Oncotarget.

[39] Casaluce F., Sgambato A., Maione P., Spagnuolo A., Gridelli C. (2018). Lung cancer, elderly and immune checkpoint inhibitors. J. Thorac. Dis..

[40] Gomes F., Woolley S., Califano R., Summers Y., Baker K., Burns K., Yorke J., Blackhall F. (2017). MA 10.07 Elderly Lung Cancer Patients on Immunotherapy: Preliminary Results from the ELDERS Study. J. Thorac. Oncol..

